# Anaemia and blood transfusion in African children presenting to hospital with severe febrile illness

**DOI:** 10.1186/s12916-014-0246-7

**Published:** 2015-02-02

**Authors:** Sarah Kiguli, Kathryn Maitland, Elizabeth C George, Peter Olupot-Olupot, Robert O Opoka, Charles Engoru, Samuel O Akech, Richard Nyeko, George Mtove, Hugh Reyburn, Michael Levin, Abdel G Babiker, Diana M Gibb, Jane Crawley

**Affiliations:** Department of Paediatrics, Mulago Hospital, Makerere University, PO Box 7070, Kampala, Uganda; Kilifi Clinical Trials Facility, KEMRI-Wellcome Trust Research Programme, PO Box 203, Nairobi, Kenya; Wellcome Trust Centre for Clinical Tropical Medicine, and Department of Paediatrics, Faculty of Medicine, Imperial College, London, W2 1PG UK; Medical Research Council Clinical Trials Unit (MRC CTU) at UCL, Aviation House, 125 Kingsway, London, WC2B 6NH UK; Department of Paediatrics, Mbale Regional Referral Hospital, Pallisa Road Zone, PO Box 921, Mbale, Uganda; Department of Paediatrics, Soroti Regional Referral Hospital, PO Box 289, Soroti, Uganda; Department of Paediatrics, St Mary’s Hospital, PO Box 180, Lacor, Uganda; Department of Paediatrics, Joint Malaria Programme, Teule Hospital, PO Box 81, Muheza, Tanzania; Centre for Tropical Medicine and Global Health, Nuffield Department of Medicine, University of Oxford, Oxford, OX3 7LD UK

**Keywords:** Africa, Anaemia, Blood transfusion, Children, FEAST trial, Malaria, Sepsis

## Abstract

**Background:**

Severe anaemia in children is a leading cause of hospital admission and a major cause of mortality in sub-Saharan Africa, yet there are limited published data on blood transfusion in this vulnerable group.

**Methods:**

We present data from a large controlled trial of fluid resuscitation (Fluid Expansion As Supportive Therapy (FEAST) trial) on the prevalence, clinical features, and transfusion management of anaemia in children presenting to hospitals in three East African countries with serious febrile illness (predominantly malaria and/or sepsis) and impaired peripheral perfusion.

**Results:**

Of 3,170 children in the FEAST trial, 3,082 (97%) had baseline haemoglobin (Hb) measurement, 2,346/3,082 (76%) were anaemic (Hb <10 g/dL), and 33% severely anaemic (Hb <5 g/dL). Prevalence of severe anaemia varied from 12% in Kenya to 41% in eastern Uganda. 1,387/3,082 (45%) children were transfused (81% within 8 hours). Adherence to WHO transfusion guidelines was poor. Among severely anaemic children who were not transfused, 52% (54/103) died within 8 hours, and 90% of these deaths occurred within 2.5 hours of randomisation. By 24 hours, 128/1,002 (13%) severely anaemic children had died, compared to 36/501 (7%) and 71/843 (8%) of those with moderate and mild anaemia, respectively. Among children without severe hypotension who were randomised to receive fluid boluses of 0.9% saline or albumin, mortality was increased (10.6% and 10.5%, respectively) compared to controls (7.2%), regardless of admission Hb level. Repeat transfusion varied from ≤2% in Kenya/Tanzania to 6 to 13% at the four Ugandan centres. Adverse reactions to blood were rare (0.4%).

**Conclusions:**

Severe anaemia complicates one third of childhood admissions with serious febrile illness to hospitals in East Africa, and is associated with increased mortality. A high proportion of deaths occurred within 2.5 hours of admission, emphasizing the need for rapid recognition and prompt blood transfusion. Adherence to current WHO transfusion guidelines was poor. The high rates of re-transfusion suggest that 20 mL/kg whole blood or 10 mL/kg packed cells may undertreat a significant proportion of anaemic children. Future evaluation of the impact of a larger volume of transfused blood and optimum transfusion management of children with Hb of <6 g/dL is warranted.

Please see related article: http://dx.doi.org/10.1186/s12916-014-0248-5.

**Electronic supplementary material:**

The online version of this article (doi:10.1186/s12916-014-0246-7) contains supplementary material, which is available to authorized users.

## Background

In sub-Saharan Africa, severe anaemia in children is a leading cause of hospital admission, a major cause of mortality [1,2], and is responsible for a high proportion of the 660,000 malaria-related deaths that are estimated to occur each year [3,4]. Although timely transfusion can be lifesaving, equitable access to adequate supplies of safe blood for transfusion remains a key challenge in sub-Saharan Africa. In order to preserve this scarce resource and to reduce the risk of transfusion-transmitted infections, guidelines developed by the World Health Organization (WHO) encourage the rational use of blood transfusion [5]. However, adherence to the guidelines is variable, and transfusion recommendations vary between countries [6-8]. There are limited published data on blood transfusion of anaemic children in sub-Saharan Africa, particularly in relation to re-transfusion [9-11]. A large randomised controlled trial of fluid resuscitation (the Fluid Expansion As Supportive Therapy (FEAST) trial [12]) provided an opportunity to present quality-controlled data on the prevalence, clinical features, and transfusion management of anaemia in children presenting to hospitals in three East African countries with serious febrile illness and clinical signs of impaired peripheral perfusion.

## Methods

The FEAST trial was conducted between January 2009 and January 2011 at six centres in three East African countries (Kenya: Kilifi District Hospital; Tanzania: Teule District Hospital, Muheza; Uganda: Mulago National Referral Hospital, Kampala; St Marys Hospital, Lacor; Soroti and Mbale Regional Referral Hospitals, Eastern Uganda). Malaria transmission at all sites in Uganda was perennial and intense, particularly in Eastern Uganda, while transmission in Kilifi and Muheza was predominantly seasonal and of low to moderate intensity during the period of the trial [13]. The study was registered on the 29^th^ of November, 2008 (ISRCTN69856593).

### Study population

Eligible children were between 60 days and 12 years of age, and presented with severe febrile illness complicated by impaired consciousness (prostration or coma) and/or respiratory distress (increased work of breathing), plus at least one sign of impaired peripheral perfusion (capillary refill time of 3 seconds or more, lower limb temperature gradient, weak radial pulse volume, or severe tachycardia (see footnotes to Table 1 for all clinical definitions).

**Table 1:** **Clinical characteristics at baseline by level of haemoglobin**

	**Severe anaemia (Hb <5 g/dL)**	**Moderate anaemia (5 to <7 g/dL)**	**Mild anaemia (7 to <10 g/dL)**	**No anaemia (≥10 g/dL)**	**Total**	***P*** **value**
N (%)^1^	1,002 (33%)	501 (16%)	843 (27%)	736 (24%)	3,082	
**General**						
Age (months); median (IQR)	25 (15, 42)	23 (13, 36)	19 (12, 34)	24 (14, 45)	24 (13, 38)	0.02
Female	439 (44%)	245 (49%)	367 (44%)	364 (49%)	1415 (46%)	0.1
Weight-for-age z-score; median (IQR)	−1.6	−1.6	−1.5	−1.3	−1.5	<0.001
	(−2.5, –0.8)	(−2.6, –0.8)	(−2.4, –0.6)	(−2.2, −0.5)	(−2.4, −0.7)	
Axillary Temperature >39°C	109 (11%)	119 (24%)	261 (31%)	234 (32%)	723 (24%)	<0.001
**Respiratory**						
Respiratory rate; mean (SD)	56.1 (13.8)	56.5 (14.5)	60.3 (15.8)	58.0 (16.6)	57.8 (15.3)	<0.001
Respiratory distress	845 (85%)	410 (82%)	694 (83%)	574 (78%)	2,523 (82%)	0.001
Chest indrawing	686 (69%)	322 (64%)	576 (68%)	483 (66%)	2,067 (67%)	0.39
Deep breathing	745 (75%)	329 (66%)	485 (58%)	424 (58%)	1,983 (65%)	<0.001
Oxygen saturation ≤90%	260 (28%)	107 (22%)	187 (23%)	195 (27%)	749 (25%)	0.36
**Cardiovascular**						
Weak radial pulse	298 (29%)	113 (23%)	139 (16%)	107 (15%)	657 (21%)	<0.001
Lower limb temperature gradient^2^	661 (66%)	295 (59%)	467 (55%)	394 (53%)	1,883 (59%)	<0.001
Capillary refill time (3 or more seconds)	573 (57%)	115 (23%)	80 (9%)	50 (7%)	818 (27%)	<0.001
Severe tachycardia^3^	616 (62%)	377 (75%)	623 (74%)	544 (74%)	2,160 (70%)	<0.001
Bradycardia	18 (2%)	3 (<1%)	6 (<1%)	8 (1%)	35 (1%)	0.11
Systolic blood pressure; median (IQR)	88 (81, 96)	93 (86, 101)	94 (86, 102)	98 (89, 106)	93 (85, 101)	<0.001
Moderate hypotension^4^	100 (10%)	25 (5%)	43 (5%)	19 (3%)	187 (6%)	<0.001
Hypothermia (temperature <36°C)	98 (10%)	24 (5%)	27 (3%)	41 (6%)	190 (6%)	<0.001
Dehydration (sunken eyes or reduced skin turgor)	105 (11%)	46 (9%)	51 (6%)	34 (5%)	236 (8%)	<0.001
**Anaemia**						
Severe pallor (pale mucous membranes)	989 (99%)	381 (77%)	167 (20%)	38 (5%)	1,575 (51%)	<0.001
Haemoglobinuria (history of dark urine)	282 (28%)	62 (12%)	32 (4%)	9 (1%)	385 (13%)	<0.001
Clinical jaundice	584 (58%)	200 (40%)	155 (18%)	35 (5%)	974 (32%)	<0.001
**Neurological**						
Prostrate^5^	726 (73%)	328 (66%)	466 (55%)	387 (53%)	1,907 (62%)	<0.001
Coma^6^	129 (13%)	89 (18%)	142 (17%)	98 (13%)	458 (15%)	0.55
Convulsions in this illness	264 (27%)	212 (42%)	358 (42%)	320 (44%)	1,154 (37%)	<0.001
**Laboratory tests**						
Positive malaria parasitaemia^7^	593 (59%)	377 (75%)	462 (55%)	317 (43%)	1,749 (57%)	<0.001
Hyperparasitaemia^8^	34/592 (6%)	41/308 (13%)	42/569 (7%)	32/504 (6%)	149/1,973 (8%)	0.86
Glucose <2.5 mmol/L	52 (5%)	28 (6%)	31 (4%)	23 (3%)	134 (5%)	0.02
Lactate ≥5 mmol/L	672 (70%)	218 (45%)	184 (23%)	75 (11%)	1,149 (39%)	<0.001
Base deficit >8	392/632 (62%)	189/329 (58%)	254/554 (45%)	239/539 (44%)	1,074/2,054 (52%)	<0.001
Severe acidaemia (pH <7.2)	97/635 (15%)	38/291 (12%)	48/556 (9%)	30/511 (6%)	213/2,061 (10%)	0.82
Hyperkalaemia (>6.5 mmol/L)	83/630 (13%)	41/330 (12%)	54/559 (10%)	28/536 (5%)	206/2,055 (10%)	<0.001
HIV antibody positive	17/790 (2%)	25/390 (6%)	36/666 (5%)	31/615 (5%)	109/2,461 (4%)	0.008
Positive blood culture	25/234 (11%)	32/179 (18%)	36/341 (11%)	35/317 (10%)	128/1,071 (12%)	0.52

^1^Of 3,170 children enrolled in the FEAST trial, 88 did not have haemoglobin measured at baseline.

^2^Temperature gradient was assessed by running the back of the hand from the toe to the knee; a positive temperature gradient was defied as a notable temperature change from cold (dorsum of foot) to warm (knee).

^3^Severe tachycardia was defined as >180 beats per minute (bpm) in children <12 months, >160 bpm in children aged 12 months to 5 years, and >140 bpm for those aged >5 years.

^4^Moderate hypotension was defined as systolic blood pressure of 50 to 75 mmHg in children younger than 12 months, 60 to 75 mmHg in those 12 months to 5 years, and 70 to 85 mmHg in those older than 5 years, as measured by automated blood pressure monitor.

^5^Prostration was defined as the inability of a child older than 8 months of age to sit upright or the inability of a child 8 months of age or younger to breast feed.

^6^Coma was defined as the inability to localise a painful stimulus.

^7^Positive on quality-controlled malaria slide, or, if missing, on rapid diagnostic test.

^8^Parasite count >250,000 per μL or, for 133 children in whom quantitative parasite count was unavailable, percentage parasitaemia >10%. No information on parasite density was available for 1,109/3,082 (36%) children.

IQR, Interquartile range; SD, Standard deviation.

### FEAST trial randomisation strategy

Children were randomised to receive immediate boluses of 20 to 40 mL/kg of 5% human albumin solution or 0.9% saline solution over one hour, or maintenance fluids at 4 mL/kg/hour until able to drink (no bolus control group). Those with severe hypotension were randomly assigned to receive 40 mL/kg bolus of either albumin or saline. Children with severe malnutrition, gastroenteritis, trauma, surgery, burns, and conditions for which volume expansion would be contraindicated were excluded from the trial. The primary endpoint was mortality at 48 hours after randomisation. Consent and ethical approval are detailed elsewhere [12]. Saline and albumin were provided by Baxter BioScience, Vienna.

### Clinical management

Venous blood was taken on admission for immediate measurement of haemoglobin (Hb), blood glucose, lactate, malaria parasitaemia, and HIV status. Hb was measured using the HaemoCue Hb 301 system (HaemoCue AB, Angelholm, Sweden), a portable point of care Hb testing kit that is factory calibrated (without need for recalibration), and using disposable microcuvettes that require no calibration between batches. The measurement range is 0 to 25.6 g/dL, and results are available in 10 seconds. Children were managed on general paediatric wards, with no facilities for ventilation other than short-term ‘bag and mask’ support. Training in triage, identification, and definitions of adverse events related to fluid management (including transfusion) were given prior to and throughout the trial, and were included in the trial manual of operations that was given to every team member.

### Blood transfusion

Clinicians at all sites followed national and WHO blood transfusion guidelines (Table 2). Children with Hb ≤4 g/dL, regardless of clinical symptoms, were transfused with 20 mL/kg whole blood or 10 mL/kg packed cells, given over 4 hours, and started as soon as possible after the first bolus of resuscitation fluid, if randomised to bolus. Children with Hb 5 to 6 g/dL were transfused if they had one or more clinical or laboratory features of severity (Table 2). Use of frusemide or other diuretics was confined to children with clinical signs of pulmonary oedema. Hb levels were checked at 8, 24, and 48 hours post-randomisation, and at any other time point if there was clinical suspicion of severe anaemia or deterioration. Repeat blood transfusion was permitted when Hb levels fell below transfusion thresholds. Any severe adverse events were reviewed by the attending clinician and subsequently reviewed by the trial oversight and ethical review committees.

**Table 2:** **Blood transfusion guidelines used by clinicians in the FEAST trial**

**Guidelines**	**Indications for transfusion**			**Volume and speed of transfusion**
	**Haemoglobin level (g/dL)**		**Clinical symptoms**	
WHO Pocket Book of Hospital Care for Children (2005)*	4 or less		Not required	20 mL/kg whole blood or 10 mL/kg packed cells over 3 to 4 hours
	OR			
	6 or less	PLUS 1 or more	Deep and laboured breathing	
			Cardiac failure	
			Clinical dehydration	
			Shock	
			Impaired consciousness	
			Malaria parasitaemia >10%	
Kenya Guidelines for Appropriate Use of Blood and Blood Products (2004)*	Less than 4		Not required	20 mL/kg whole blood over 3 to 4 hours
	OR			
	Less than 5	PLUS	Respiratory distress	
Ugandan National Guidelines (2010)*	4 or less		Not required	20 mL/kg whole blood or 10 mL/kg packed cells over 3 to 4 hours
	OR			
	6 or less	PLUS 1 or more	Hypoxia	
			Cardiac decompensation	
			Acidosis	
			Impaired consciousness	
			Cerebral malaria	
			Septicaemia	
			Meningitis	
			Malaria parasitaemia >20%	
Tanzania National Malaria Guidelines (2006)*	4 or less		Not required	20 mL/kg whole blood or 10 mL/kg packed cells over 3 to 4 hours
	OR			
	6 or less	PLUS	Cardiac failure	

*Version of guidelines available at the time of the FEAST trial.

### Statistical analysis

**Figure 1:**
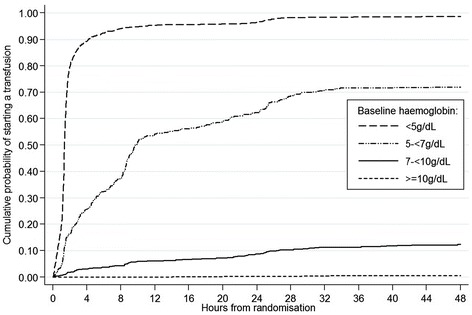
**Kaplan Meier curves of time to first transfusion, by anaemia category.**

**Figure 2:**
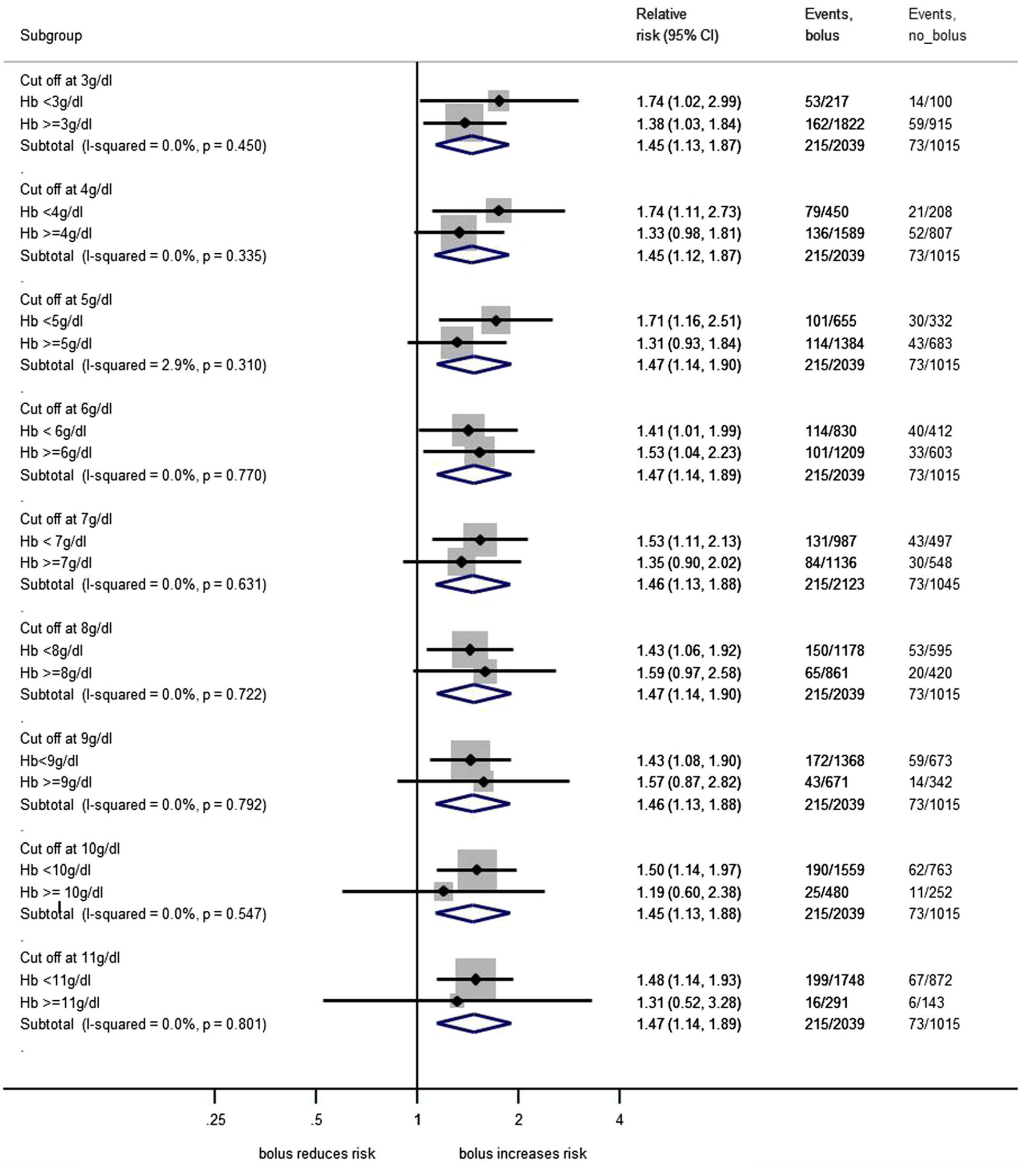
**Forest plot comparing 48-hour mortality in fluid bolus and control groups, by admission haemoglobin level.**

**Figure 3:**
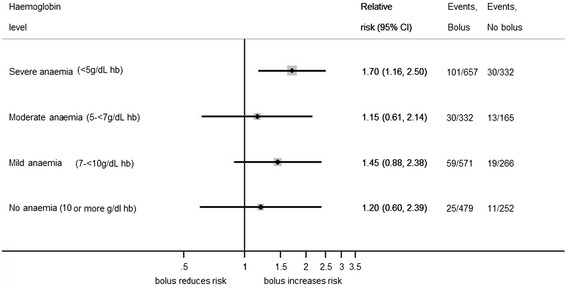
**Forest plot comparing 48-hour mortality in fluid bolus and control groups, by anaemia category.**

Children were classified as having severe anaemia (Hb <5 g/dL), moderate anaemia (Hb 5 to <7 g/dL), mild anaemia (Hb 7 to <10 g/dL), or no anaemia (Hb ≥10 g/dL) on the basis of admission Hb levels (Table 1). Baseline characteristics were compared across all groups using the *χ*^2^ test for trend across categorised variables, non-parametric test of medians across ordered groups, and linear regression for comparison of means. The prevalence of anaemia, jaundice, and malaria, number of blood transfusions, and mortality at 48 hours and 28 days were summarised by site. Kaplan-Meier graphs were plotted, showing time from randomisation to the start of blood transfusion by baseline Hb level (Figure 1) and by site (Additional file 1: Figure S1), with children censored at the time transfusion started, time of death, or 48 hours after randomisation (whichever occurred first). Inter-site differences were examined with the log-rank test. Forest plots were constructed to compare 48-hour mortality in the fluid bolus and control groups according to Hb level (Figure 2) and anaemia category (severe, moderate, mild, or no anaemia; Figure 3) at the time of admission.

Although WHO guidelines recommend transfusion for all children with Hb ≤4 g/dL, guidance is less clear in relation to children with Hb of 5 to 6 g/dL, for whom transfusion is only recommended in the presence of one or more pre-specified clinical and laboratory features [5]. To assess adherence to WHO guidelines within the FEAST trial, we therefore examined the factors most strongly associated with blood transfusion in children with Hb ≥5 g/dL. A time-updated Cox regression model for time to first transfusion was fitted for those with baseline Hb of ≥5 g/dL. Regression models were first calculated for each risk factor individually, adjusting for randomisation arm, Hb at baseline, and most recent Hb at 8 or 24 hours. A multivariable, time-updated Cox regression model, similarly adjusted, was then constructed by stepwise backwards elimination, including study site and clinical indications for transfusion, where available (Table 3). Using variables found to be risk factors for time to first transfusion, multivariable logistic regression, adjusted for randomisation arm, was used to describe baseline risk factors for re-transfusion. Sub-group analyses by Hb level and blood transfusion were pre-specified, while other analyses were performed *post hoc*.

**Table 3:** **Clinical predictors of transfusion in children with admission Hb ≥5 g/dL**

		**Univariable analyses** ^**1**^		**Multivariable analyses (n = 1,962)**	
**Variable**	**Number in univariate model**	**HR (95% CI)**	***P*** **value** ^**2**^	**HR (95% CI)**	***P*** **value** ^**2**^
Updated (most recent) haemoglobin (per 1 g/dL increase)		–	–	0.46 (0.41–0.52)	<0.001
Haemoglobin at admission (per 1 g/dL increase)		–	–	0.84 (0.74–0.95)	0.005
Severe pallor	2,076	3.21 (2.47–4.19)	<0.001	3.18 (2.42–4.19)	<0.001
Jaundice (clinical)	2,079	1.57 (1.30–1.89)	<0.001	1.27 (0.97–1.68)	0.08
Haemoglobinuria	2,078	1.69 (1.28–2.21)	<0.001	–	–
*P. falciparum* malaria	2,072	1.42 (1.13–1.80)	0.003	–	–
Weak pulse volume	2,080	1.37 (1.10–1.70)	0.005	–	–
Severe tachycardia	2,079	0.88 (0.71–1.10)	0.26	0.80 (0.63–1.00)	0.054
Capillary refill time ≥3 s	2,079	1.58 (1.27–1.97)	<0.001	1.59 (1.24–2.03)	0.001
Temperature gradient	2,080	1.30 (1.07–1.58)	0.007	–	–
Respiratory distress	2,072	1.23 (0.96–1.58)	0.09	–	–
Indrawing	2,080	1.24 (1.02–1.51)	0.04	1.30 (1.01–1.69)	0.04
Deep breathing	2,080	1.24 (1.07–1.44)	0.005	–	–
Dehydration	2,078	1.27 (0.93–1.74)	0.14	1.33 (0.95–1.85)	0.09
Lactate at admission (per 1 mmol/L increase)	1,970	1.09 (1.07–1.17)	<0.001	1.08 (1.05–1.10)	<0.001
Oxygen saturation (per 1% increase)	2,047	1.00 (0.99–1.02)	0.91	–	–
Consciousness level	2,078		0.22	–	–
Alert		1 (ref)		–	–
Prostration		1.26 (0.97–1.65)	0.09	–	–
Coma		1.22 (0.88–1.68)	0.24	–	–
Site	2,080		<0.001		<0.001
Mbale, Uganda		1 (ref)		1 (ref)	
Kilifi, Kenya		0.44 (0.27–0.71)	0.001	0.38 (0.21–0.68)	0.001
Mulago, Uganda		0.91 (0.71–1.16)	0.44	0.98 (0.70–1.40)	0.93
Soroti, Uganda		1.18 (0.91–1.54)	0.21	1.39 (1.00–1.96)	0.05
Lacor, Uganda		0.63 (0.44–0.88)	0.008	0.71 (0.46–1.11)	0.13
Teule, Tanzania		1.35 (0.90–2.05)	0.15	1.56 (0.96–2.54)	0.08

^1^All models adjusted for baseline haemoglobin, most recent haemoglobin, and randomisation arm.

^2^ *P* value for Likelihood ratio test overall or Wald test for categories.

Bacteraemia was not included in the model as only 1,071 children had a blood culture.

### Study oversight

The ethics committees at Imperial College, London, Makerere University, Uganda, Medical Research Institute, Kenya, and National Medical Research Institute, Tanzania, approved the protocol. In cases in which prior written consent from parents of guardians could not be obtained, provision was made for oral assent from a legal surrogate, followed by delayed written informed consent as soon as practicable. Initial boluses were increased to from 20 to 40 mL/kg (and 40 to 60 mL/kg in those with severe hypotension) in a protocol amendment dated August 2010 and approved by all ethics committees.

## Results

### Prevalence and clinical presentation of anaemia

Of 3,170 children in the FEAST trial, 3,082 (97%) had a baseline Hb measurement, and 2,346/3,082 (76%) were anaemic (Table 1); 1,002/3,082 (33%) children had severe anaemia, 501 (16%) moderate anaemia, and 843 (27%) mild anaemia. Children with moderate or severe anaemia had worse nutritional status than those with mild anaemia or no anaemia. A greater proportion of severely anaemic children had impaired peripheral perfusion, moderate hypotension, low core temperature, dehydration, respiratory distress, and prostration compared to those with mild or moderate anaemia and non-anaemic children (*P* ≤0.001 for all comparisons). The proportion of children with oxygen saturation ≤90% did not differ significantly between groups. Severe pallor, jaundice, dark urine, metabolic acidosis, and hyperkalaemia were more common in children with severe anaemia (*P* ≤0.001 for all comparisons). Malaria parasitaemia was present in 1,749/3,082 (57%) children, with a higher proportion of anaemic children being parasitaemic compared to non-anaemic children (59%, 75%, and 55% for severe, moderate, and mild anaemia, respectively, versus 43% for no anaemia; *P* <0.001). Among those children in whom malaria parasitaemia was quantified, 149/1,973 (8%) had hyperparasitaemia, with no significant differences between anaemic and non-anaemic children. Overall, 128/1,071 (12%) blood cultures were positive, with no significant difference between groups; 109/2,461 (4%) children tested HIV antibody positive.

The prevalence of severe anaemia was lowest in Kilifi, Kenya (13%), and highest in Mbale (39%) and Soroti (43%), Eastern Uganda (Table 4). Haemoglobinuria was uncommon (≤2%) in Kenya and Tanzania, but occurred in ~20% of children presenting to Mbale and Soroti, often in association with jaundice (Table 4).

**Table 4:** **Prevalence of anaemia, number of blood transfusions and mortality, by site**

	**Kenya**	**Uganda**				**Tanzania**	**TOTAL**
**N (%)**	**Kilifi**	**Mulago**	**Soroti**	**Lacor**	**Mbale**	**Teule**	
Children with admission Hb	215	745	629	232	1,164	97	3,082
**ADMISSION FINDINGS**							
Severe pallor	77 (36%)	310 (42%)	364 (58%)	122 (53%)	648 (56%)	54 (56%)	1,575 (51%)
Jaundice	1 (<1%)	55 (7%)	169 (27%)	27 (12%)	721 (62%)	1 (1%)	974 (32%)
Haemoglobinuria	1 (<1%)	46 (6%)	136 (22%)	26 (11%)	174 (15%)	2 (2%)	385 (13%)
Malaria parasitaemia	87 (40%)	369 (50%)	338 (54%)	160 (69%)	742 (64%)	53 (55%)	1,749 (57%)
**BLOOD TRANSFUSION**							
**All children**							
Received any transfusion (%)	45 (21%)	251 (34%)	332 (53%)	97 (42%)	616 (53%)	46 (47%)	1,387 (45%)
Received one or more re-transfusions (% of those transfused)	1 (2%)	82 (33%)	68 (20%)	14 (14%)	150 (24%)	2 (4%)	317 (23%)
**Severe anaemia (Hb <5 g/dL)**							
At admission	28 (13%)	172 (23%)	269 (43%)	59 (25%)	452 (39%)	22 (23%)	1,002 (33%)
At 8 hours*	15/193 (8%)	93/704 (13%)	90/534 (17%)	40/204 (20%)	174/1,078 (16%)	16/83 (19%)	428/2,796 (15%)
At 24 hours*	13/191 (7%)	46/687 (7%)	59/568 (10%)	31/197 (16%)	89/1,032 (9%)	4/79 (5%)	242/2,754 (9%)
Received any transfusion (%)	26 (93%)	156 (91%)	250 (93%)	56 (95%)	425 (94%)	20 (91%)	933 (94%)
Received one or more re-transfusions (% of those transfused)	1 (4%)	65 (42%)	61 (24%)	14 (25%)	133 (31%)	1 (5%)	275 (29%)
**Moderate anaemia (Hb 5 to <7 g/dL)**							
At admission	32 (15%)	110 (15%)	81 (13%)	51 (22%)	204 (18%)	23 (24%)	501 (16%)
At 8 hours*	39/193 (20%)	191/704 (27%)	185/534 (35%)	68/204 (33%)	326/1,078 (30%)	27/83 (33%)	836/2,796 (30%)
At 24 hours*	39/191 (20%)	163/687 (24%)	182/568 (32%)	71/197 (36%)	329/1,032 (32%)	20/79 (25%)	804/2,754 (29%)
Received any transfusion (%)	16 (50%)	76 (69%)	58 (72%)	30 (59%)	150 (74%)	21 (91%)	351 (70%)
Received one or more re-transfusions (% of those transfused)	0 (0%)	17 (22%)	7 (12%)	0 (0%)	16 (11%)	1 (5%)	41 (12%)
**Mild anaemia (Hb 7 to <10 g/dL)**							
At admission	88 (41%)	198 (27%)	148 (24%)	83 (36%)	301 (26%)	25 (26%)	843 (27%)
At 8 hours*	106/193 (55%)	267/704 (27%)	210/534 (39%)	75/204 (37%)	429/1,078 (39%)	32/83 (39%)	1,119/2,796 (40%)
At 24 hours*	105/191 (55%)	309/687 (45%)	239/568 (42%)	76/197 (39%)	474/1,032 (46%)	41/79 (52%)	1,244/2,754 (45%)
Received any transfusion (%)	3 (3%)	19 (10%)	22 (15%)	11 (13%)	40 (13%)	4 (16%)	99 (12%)
Received one or more transfusions (% of those transfused)	0 (0%)	0 (0%)	0 (0%)	0 (0%)	1 (3%)	0 (0%)	1 (1%)
**MORTALITY**							
Died by 48 hours	21 (10%)	62 (8%)	58 (9%)	36 (16%)	108 (9%)	20 (21%)	305 (10%)
Died by 28 days	26 (12%)	76 (10%)	70 (11%)	41 (18%)	118 (10%)	22 (23%)	353 (12%)

*Denominators vary due to deaths or missing Hb values.

### Blood transfusion

#### Number and type of transfusion

Overall, 1,387/3,082 (45%) children were transfused, of whom 23% (317/1,387) required re-transfusion; 81% (1,118/1,387) were transfused within 8 hours of admission. The proportion of children without severe hypotension transfused in each of the three randomised arms was similar (468/1,024 (46%), 470/1,015 (46%), and 438/1,015 (43%) in the albumin bolus arm, saline bolus arm, and no bolus arm, respectively). Of the 28/3,082 children presenting with severe hypotension who were separately randomised to receive albumin or saline bolus, 11 (38%) were also transfused.

In total, 94% (933/1,002) of severely anaemic children were transfused, with 275/933 (29%) receiving more than two transfusions, this proportion varying from ≤5% in Kenya and Tanzania to between 23% and 38% in the four Ugandan centres; 70% (351/501) with moderate anaemia and 12% (99/843) with mild anaemia were transfused, of whom 41 (8%) children with moderate anaemia and one child with mild anaemia received two or more transfusions.

Whole blood was used for 1,459/1,767 (83%) transfusions, and packed red blood cells, which were only available at Mulago Hospital, for 308/1,767 (17%) transfusions. Five transfused children received frusemide during their clinical course in hospital.

#### Timing and indications for transfusion and re-transfusion

Among surviving severely anaemic children, 94% were transfused by 8 hours, 96% by 24 hours, and 99% by 48 hours of admission (Figure 1). The proportion of moderately anaemic children who were transfused increased rapidly during the first 2 hours of admission, and again between 8 and 10 hours, following repeat Hb measurement at 8 hours. The proportion of children with moderate anaemia who were transfused at each time point varied significantly (*P* <0.001) between centres (Additional file 1: Figure S1). Among 843 children who were mildly anaemic on admission, 4% were transfused by 8 hours, and 8% by 24 hours. Four children with baseline Hb ≥10 g/dL were transfused during the first 48 hours of admission when their Hb level fell <6.5 g/dL.

A Cox regression model for time to first transfusion in children with baseline Hb ≥5 g/dL and complete information on clinical predictors of transfusion (n = 1,962, 428 transfused; Table 3) showed that although most recent Hb level was a very strong clinical predictor of transfusion, Hb at baseline, severe pallor, jaundice, capillary refill time ≥3 seconds, lactate, and chest indrawing were independent predictors. Children were more likely to be transfused in Soroti and Teule compared to Mbale (the largest site, used as the reference), although Mbale had the highest proportion (13%) of children receiving two or more transfusions. Although malaria parasitaemia, temperature gradient, weak pulse, deep breathing, and respiratory distress were associated with time to transfusion in univariable models, they were not significantly associated in the multivariable model. Severe tachycardia, dehydration, prostration, coma and oxygen saturation were not significantly associated with time to first transfusion in either analysis. There was no evidence of an association between randomisation arm and risk of subsequent transfusion.

Factors predictive of re-transfusion among children who were transfused at least once (n = 1,387) were admission Hb, coma, pallor, and severe tachycardia (Table 5). Even when controlling for baseline Hb level, children at Mulago hospital were twice as likely to be re-transfused compared to the other sites.

**Table 5:** **Clinical predictors of re-transfusion in children transfused at least once (n = 1,387)**

**Admission variable**	**Odds ratio (95% CI)**	***P*** **value**
Haemoglobin at admission (per 1 g/dL increase)	0.68 (0.60–0.76)	<0.001
Lactate at admission (per 1 mmol/L increase)	1.01 (0.98–1.05)	0.57
**Consciousness level**		
Prostration (inability to sit or breastfed if <8 months)	0.94 (0.62–1.44)	0.77
Coma (inability to localise a painful stimulus)	0.25 (0.12–0.51)	<0.001
Clinical jaundice	1.42 (0.98–2.06)	0.06
Indrawing	0.97 (0.66–1.41)	0.86
Deep breathing (Kussmaul’s breathing)	1.30 (0.92–1.83)	0.14
Capillary refill time ≥3 s	1.08 (0.78–1.49)	0.65
Dehydration	1.64 (1.03–2.61)	0.04
Severe pallor*	14.4 (1.88–104.9)	0.01
Severe tachycardia	0.69 (0.51–0.93)	0.02
**Site**		
Mbale, Uganda	1 (ref)	
Kilifi, Kenya	0.15 (0.02–1.21)	0.08
Mulago, Uganda	2.36 (1.42–3.92)	0.001
Soroti, Uganda	0.82 (0.52–1.29)	0.39
Lacor, Uganda	0.84 (0.40–1.75)	0.64
Teule, Tanzania	0.29 (0.06–1.35)	0.12

*Only 7% of the children included in this model did not have pallor at baseline and there are a small number of events in this group, giving a wide confidence interval.

#### Outcome of transfusion

Among 899 children who were severely anaemic at baseline and transfused within 8 hours of admission, 8-hour Hb level remained <5 g/dL in 227 (26%) and ≥5 g/dL in 591 (66%), while 39 (4%) children had died (Table 6). For 103 severely anaemic children who were not transfused in the first 8 hours, Hb level at 8 hours remained <5 g/dL in 26 (25%) and ≥5 g/dL in 20 (19%), while 54 (52%) children had died; 90% of these deaths occurred within 2.5 hours of randomization and 100% within 5 hours. Overall, 67% (93/139) of deaths among severely anaemic children occurred within 8 hours of admission and 92% (128/139) by 24 hours. For 70% (89/128) of the severely anaemic children who died within 24 hours, mode of death was considered by an independent Endpoint Review Committee, who were blind to the randomised arm, to be cardiogenic (defined as signs of shock, severe tachycardia or bradycardia plus one of prolonged capillary refill time ≥3 seconds, cold peripheries, or low blood pressure at the point of demise), and indicative of myocardial dysfunction rather than biventricular failure [14]. Among 184 children with moderate anaemia at baseline who were transfused within the first 8 hours, Hb level at 8 hours fell to <5 g/dL in 15 (8%) and was ≥5 g/dL in 148 (80%), while 10 (5%) children had died. For the 317 children with moderate anaemia who were not transfused by 8 hours, Hb at 8 hours had fallen to <5 g/dL in 107 (34%) and was ≥5 g/dL in 184 (58%), while 12 (4%) had died. Of the 35 children with mild anaemia who were transfused within the first 8 hours of admission, Hb level at 8 hours had fallen to <5 g/dL in 2 (6%) and was ≥5 g/dL in 23 (66%), while 8 (23%) had died. Among the 808 children with mild anaemia at baseline who were not transfused by 8 hours, 8-hour Hb level was <5 g/dL in 14 (2%) and ≥10 g/dL in 685 (85%), while 29 (4%) had died.

**Table 6:** **Mortality and haemoglobin level at 8 and 24 hours by transfusion and baseline anaemia status**

	**Anaemia status at baseline**				
	**Severe**	**Moderate**	**Mild**	**No anaemia**	**Total**
	**Hb <5 g/dL**	**Hb 5 to <7 g/dL**	**Hb 7 to <10 g/dL**	**Hb ≥10 g/dL**	
**Status at 8 hours**	1,002	501	843	736	3,082
**TRANSFUSED**	899	184	35	0	1,118
Hb <5 g/dL	227 (26%)	15 (8%)	2 (6%)	0 (0%)	244 (22%)
Hb 5 to <7 g/dL	347 (39%)	57 (31%)	4 (11%)	0 (0%)	408 (37%)
Hb 7 to <10 g/dL	227 (25%)	79 (43%)	16 (46%)	0 (0%)	322 (29%)
Hb ≥10 g/dL	17 (2%)	12 (7%)	3 (9%)	0 (0%)	32 (3%)
Hb missing – as died before 8 hours	39 (4%)	10 (5%)	8 (23%)	0 (0%)	57 (5%)
Hb missing – other reason	42 (5%)	11(6%)	2 (6%)	0 (0%)	54 (5%)
**NOT TRANSFUSED**	103	317	808	736	1,964
Hb <5 g/dL	26 (25%)	107 (34%)	14 (2%)	1 (<1%)	148 (8%)
Hb 5 to <7 g/dL	7 (7%)	51 (16%)	39 (5%)	2 (<1%)	99 (5%)
Hb 7 to <10 g/dL	4 (4%)	6 (2%)	13 (2%)	1 (<1%)	24 (1%)
Hb ≥10 g/dL	9 (9%)	128 (40%)	685 (85%)	683 (93%)	1505 (77%)
Hb missing – as died before 8 hours*	54 (52%)	12 (4%)	29 (4%)	22 (3%)	117 (6%)
Hb missing – other reason	3 (3%)	13 (4%)	28 (3%)	27 (4%)	71 (4%)
**Status at 24 hours**	1,002	501	843	736	3,082
**TRANSFUSED**	919	305	71	2	1,297
Hb <5 g/dL	143 (16%)	20 (7%)	7 (10%)	0 (0%)	170 (13%)
Hb 5 to <7 g/dL	361 (39%)	96 (31%)	12 (17%)	0 (33%)	469 (36%)
Hb 7 to <10 g/dL	293 (32%)	141 (46%)	29 (41%)	2 (67%)	465 (36%)
Hb ≥10 g/dL	31 (3%)	21 (7%)	8 (11%)	0 (0%)	60 (5%)
Hb missing – as died before 24 hours	71 (8%)	19 (6%)	13 (18%)	0 (0%)	103 (8%)
Hb missing – other reason	20 (2%)	8 (3%)	2 (3%)	0 (0%)	30 (2%)
**NOT TRANSFUSED**	83	196	772	734	1,785
Hb <5 g/dL	10 (12%)	26 (13%)	8 (1%)	0 (0%)	44 (2%)
Hb 5 to <7 g/dL	5 (6%)	12 (6%)	17 (2%)	2 (<1%)	36 (2%)
Hb 7 to <10 g/dL	3 (4%)	4 (2%)	5 (<1%)	0 (0%)	12 (<1%)
Hb ≥10 g/dL	7 (8%)	131 (67%)	665 (86%)	674 (92%)	1,477 (83%)
Hb missing – as died before 24 hours	57 (69%)	17 (3%)	58 (8%)	32 (4%)	164 (9%)
Hb missing – other reason	1 (1%)	6 (3%)	19 (2%)	26 (4%)	52 (3%)

*90% of these children died within 2.5 hours of randomisation.

By 24 hours, 128/1,002 (13%) severely anaemic children had died, compared to 36/501 (7%) moderately anaemic and 71/843 (8%) mildly anaemic children. Mortality at 48 hours for children with a baseline Hb measurement was 305/3,082 (10%), with 267/305 (88%) of all deaths occurring within 24 hours of admission. Among the 3,054 children with a baseline Hb measurement but without severe hypotension on admission, mortality in those randomised to receive fluid boluses of 0.9% saline or albumin was increased (10.6% and 10.5%, respectively), compared to no-bolus controls (7.2%), regardless of admission Hb level, and with no evidence of heterogeneity (*P* >0.3) between any subgroups (Figures 2 and 3).

#### Blood transfusion reactions

Following blinded adjudication by the trial Endpoint Review Committee, 6 of the 1,387 children in receipt of one or more transfusions (0.4%) were considered to have had a “probable” blood transfusion reaction. These children all developed fever and an urticarial rash 1 to 3 hours after the start of blood transfusion. In all cases, the transfusion was discontinued and the child treated with intravenous hydrocortisone, with or without antihistamines. All children recovered fully.

## Discussion

We have used data from a large randomised clinical trial of fluid resuscitation strategies to describe the prevalence, clinical features, and blood transfusion management of anaemia in children presenting to six hospitals in malaria-endemic regions of Uganda, Kenya, and Tanzania with severe febrile illness and clinical signs of impaired peripheral perfusion. The majority (76%) of the 3,082 children in this study were anaemic on admission, while one third (33%) were severely anaemic. The prevalence of severe anaemia varied from 12% in Kenya to between 23 to 43% in Uganda, where an increased proportion of children presented with jaundice, severe pallor, and haemoglobinuria. Almost half of all patients (including 94% of those with severe anaemia) were transfused, while 10% received two or more transfusions. The frequency of repeat transfusion varied between sites, from ≤2% in Kenya and Tanzania to between 6% and 13% at the four sites in Uganda. Adherence to WHO transfusion guidelines was poor. There was no evidence of an association between randomisation arm and risk of subsequent transfusion. Adverse reactions to blood were rare (0.4%). In-patient mortality at 48 hours was 10%, while 91% of all deaths (92% among severely anaemic children) occurred within 24 hours of admission. Among severely anaemic children who were not transfused, 52% (54/103) had died by 8 hours, and 90% of these deaths occurred within 2.5 hours of randomisation. Mortality among children randomised to receive fluid boluses with saline or albumin was significantly increased by 3.3% compared to controls, regardless of admission Hb level.

Globally, the prevalence of anaemia and severe anaemia is highest among young children in low- and middle-income regions [1]. Although micronutrient deficiencies, infectious and parasitic diseases, and haemoglobinopathies contribute to the multifactorial aetiology of severe childhood anaemia [15], malaria plays an important role in endemic areas [16]. The prevalence of severe anaemia in our study sites was lowest in Kilifi, Kenya, where malaria transmission is seasonal and of intermediate intensity, and highest in Mbale and Soroti (Eastern Uganda), where malaria transmission is perennial and intense [13]. The prevalence of confirmed bacteraemia (sepsis) was similar across all anaemia categories.

To be eligible for the FEAST trial, children had to have respiratory distress or impaired consciousness, plus one or more signs of impaired perfusion. “Respiratory distress” is a term used to describe the subjective clinical impression of increased work of breathing. Historically, respiratory distress in children with severe malarial anaemia has been considered synonymous with congestive cardiac failure [17], though subsequent work suggests that metabolic acidosis [18,19] and hypovolaemia [20-22] may be central to the pathophysiology. The increased proportion of severely anaemic children in this study with clinical signs of dehydration, impaired peripheral perfusion, hypotension, and severe lactic acidosis supports this hypothesis. In contrast, the prevalence of chest indrawing, an accepted feature of acute lower respiratory tract infections [5], was similar across each of the anaemic and non-anaemic groups. Mortality of hospitalised children with asymptomatic severe malarial anaemia is ~1%, increasing to 35% when associated with deep breathing and/or lactic acidosis and impaired consciousness [23,24].

Clinicians participating in the FEAST trial were free to follow WHO and national transfusion guidelines. WHO guidelines (on which most national guidelines are based) advise transfusion for all children whose Hb level is 4 g/dL or less, regardless of concomitant symptoms. Transfusion guidance for children with Hb of 5 to 6 g/dL is less clear-cut, since it depends upon the presence of one or more additional clinical or laboratory criteria, for which the evidence base is limited. Several of these criteria (“shock”, “clinical dehydration”, “impaired consciousness”) are not defined in the guidelines, while others (“cardiac failure”) cannot be accurately diagnosed at the bedside. Malaria parasitaemia (“malaria parasitaemia >10%”) is not routinely quantified in many hospitals throughout Africa. When guidance is ambiguous, or in situations where clinicians lack confidence in the accuracy of Hb results produced by their local laboratory [25], their decision to transfuse may be based on a subjective assessment of clinical severity [26]. Our multivariable Cox regression model suggests that transfusion of children with Hb ≥5 g/dL was associated with pallor, prolonged capillary refill time, lactate, and chest indrawing, but not with malaria parasitaemia, deep breathing, respiratory distress, prostration, coma, or dehydration. Pallor is an unreliable predictor of anaemia in hospital-based studies [27]. There is an urgent need to obtain robust evidence on which clinical criteria (if any) should be used to guide transfusion of children with Hb levels of 5 to 6 g/dL [28].

As far as we are aware, this is the first report to document the frequency of repeat blood transfusion among patients with severe anaemia. Nearly half (45%) of all children in this study were transfused, while 23% of transfused children required re-transfusion. Children presenting with severe anaemia in Uganda were 5 to 8 times more likely to receive a second transfusion than children in Kenya or Tanzania, likely reflecting lower admission Hb levels (Table 4) and an increased proportion of children (particularly in Mbale and Soroti) whose clinical presentation suggested intravascular haemolysis, highlighting the importance of repeat Hb measurement after blood transfusion, particularly in children with malaria parasitaemia [10,29].

The highest proportion of repeat transfusions occurred in Mulago hospital, Uganda (Table 4), the only site in which packed red cells were routinely available. Although this may reflect the increased ease of access to blood banking facilities within this tertiary level teaching hospital, it is possible that transfusion with 10 mL/kg of packed red cells provided inadequate treatment for children with severe anaemia.

The high proportion of repeat transfusions also suggests that 20 mL/kg whole blood may be insufficient for children with severe anaemia and ongoing haemolysis. Current WHO transfusion guidelines [30] recommend 20 mL/kg of whole blood or 10 mL/kg packed cells for children with Hb ≤6 g/dL, yet standard calculations indicate that this under-treats children with profound anaemia (Hb <4 g/dL) by ~30% [31] Other researchers have shown a modest rise in Hb of 2.5 to 3.3 g/dL following initial transfusion of 20 mL/kg [10,29,32], with post-transfusion Hb remaining <5 g/dL in ~25% of severely anaemic children [10], as we found here. Multiple, low volume (20 mL/kg of whole blood) transfusions are wasteful, inefficient, and may expose children to an increased risk of transfusion reactions and blood-borne infection. Recent evidence suggests that transfusion of children with Hb <6 g/dL (median Hb 4.2 g/dL; interquartile range, 3.1 to 4.9) with 30 mL/kg of whole blood or 15 mL/kg of packed red cells is safe, and is associated with accelerated haematological recovery [33]. This needs to be confirmed in a larger number of children, including those with Hb <3 g/dL, who may be compromised by the cardio-depressant effects of profound metabolic acidosis.

Although rapid blood transfusion in children with severe decompensated anaemia can be lifesaving, the majority of hospitals in sub-Saharan African have inadequate blood banking facilities and transfusion may be delayed until a donor is found [2]. Consequently, ~60% of deaths among children with severe malarial anaemia occur within 4 to 6 hours of admission, often before transfusion can be administered [9,10]. In our study, 90% of severely anaemic children were transfused during the first 8 hours of admission, reflecting the efficiency of local transfusion services. By 8 hours, 4% (39/899) of these children had died, compared to 52% (54/103) of those who were not transfused; 90% of these deaths occurred within 2.5 hours of randomization, highlighting the critical importance of prompt transfusion. In contrast, 8-hour mortality of children with mild anaemia was higher among transfused children (8/35, 23%) than in those who were not transfused (29/808, 4%), suggesting (though numbers are small) that clinical signs of severe illness, unrelated to anaemia, may have prompted the decision to transfuse. Confining blood transfusion to children with moderate or severe anaemia would probably make better use of a scarce commodity, and might have averted some of the deaths that occurred among the severely anaemic children who were still awaiting transfusion at 8 hours.

The data presented here were obtained within the context of a large randomised trial of fluid resuscitation strategies for children with severe febrile illness and signs of impaired perfusion. Our study population therefore represents the most severe end of the spectrum of anaemic children presenting to hospital admission in sub-Saharan Africa and concomitant morbidity and mortality may consequently have been over-represented.

There has been much debate over whether the increased mortality that was observed in the FEAST trial among children randomised to receive fluid boluses of 0.9% saline or albumin was due to haemodilution of children who were already compromised by critically low levels of Hb [34-36] . However, mortality of children who received fluid boluses was increased at all levels of admission Hb (Figure 2), with no evidence of heterogeneity between subgroups, strongly suggesting that this was not the case.

## Conclusions

We have described the prevalence, clinical features, and blood transfusion management of a large group of severely ill children admitted to six hospitals in East Africa. Our data confirm the importance of rapidly identifying sick, severely anaemic children and ensuring that they are promptly transfused. They also suggest that adherence to current WHO transfusion guidelines, even within the context of a clinical trial, is sub-optimal, and that transfusion with 20 mL/kg whole blood or 10 mL/kg packed cells may undertreat a significant proportion of anaemic children. A clinical trial to evaluate the impact of using a larger volume of transfused blood and determine the optimum transfusion management of children with Hb of <6 g/dL is underway (TRACT trial: ISRCTN84086586).

## Additional file

Additional file 1: Figure S1. Kaplan Meier curves of time to first transfusion, by site and anaemia category.

## Abbreviations

FEAST: The Fluid Expansion As Supportive Therapy trial
Hb: Haemoglobin
WHO: World Health Organization
