# Supplementary figures and images for: Anaemia and blood transfusion in African children presenting to hospital with severe febrile illness
Source: BMC Med. 2015 Feb 2;13:21. doi: 10.1186/s12916-014-0246-7 (PMC4313469; doi:10.1186/s12916-014-0246-7)

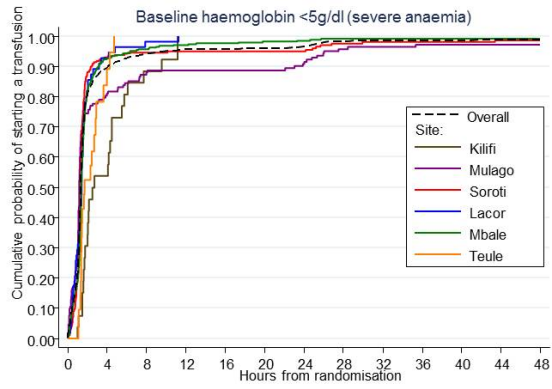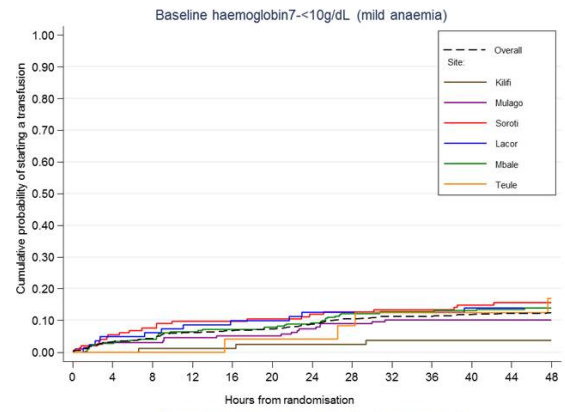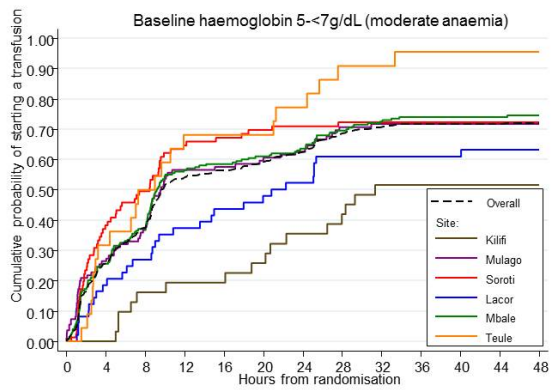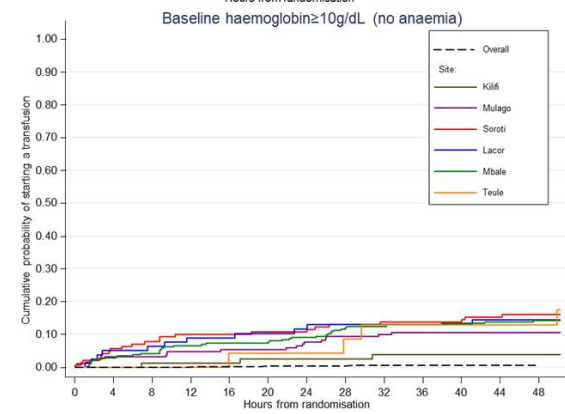

Supplement: Additional file 1: Figure S1. — Kaplan Meier curves of time to first transfusion, by site and anaemia category. [file 12916_2014_246_MOESM1_ESM.pdf]
